# Lactylation as a cross-regulatory node in tumor metabolism and epigenetics: insights and therapeutic implications

**DOI:** 10.3389/fimmu.2025.1675480

**Published:** 2025-09-25

**Authors:** Zhuangwei Lv, Ruohao Yang, Lulu Liu, Xiaoyu Shi, Ruihan Wang, Jinhua Wu, Junna Jiao

**Affiliations:** ^1^ School of Forensic Medicine, Xinxiang Medical University, Xinxiang, China; ^2^ School of Basic Medical Sciences, Xinxiang Medical University, Xinxiang, China; ^3^ School of Junji College, Xinxiang Medical University, Xinxiang, Henan, China; ^4^ Xinxiang Engineering Technology Research Center of Immune Checkpoint Drugs for Liver-Intestinal Tumors, Xinxiang Medical University, Xinxiang, China

**Keywords:** lactylation, metabolic reprogramming, epigenetic regulation, Warburg effect, therapeutic

## Abstract

Lactylation serves as a pivotal cross-regulatory mechanism linking tumor metabolic reprogramming and epigenetic regulation. This review comprehensively summarizes the mechanisms of lactylation writers, erasers, and readers, highlighting their tumor-specific functions, roles in immunosuppressive tumor microenvironment (TME) remodeling, and contributions to therapeutic resistance. Emerging targeting strategies, including metabolic inhibitors, epigenetic modulators, and combination immunotherapies, exhibit promising preclinical efficacy, highlighting their potential for clinical translation in overcoming therapy resistance and improving cancer immunotherapy.

## 1 Introduction

The crosstalk between tumor metabolic reprogramming and epigenetic regulation represents a core focus in contemporary cancer research. The landmark discovery of lysine lactylation (Kla) in 2019 provided a breakthrough in this field, revealing a direct molecular link between lactic acid—a classic end-product of glycolysis—and the regulation of gene expression (1–7). Previously regarded primarily as a metabolic waste product, lactic acid, through lactylation modification, has emerged as a key molecule connecting aberrant tumor metabolism to epigenetic remodeling (8). This post-translational modification plays a central role in tumorigenesis, progression, immune evasion, and therapeutic resistance by transducing cellular metabolic status into heritable gene expression patterns (1, 9–13).

Accumulating evidence confirms that lactylation is abnormally activated across diverse tumor types, with its levels closely correlating with tumor malignancy, patient prognosis, and therapeutic response (14). Mechanistically, lactylation regulates downstream target gene transcription via histone modification or alters protein function through non-histone modification, thereby participating in critical biological processes such as tumor cell proliferation, apoptosis, invasion, and metastasis (15). Concurrently, lactic acid accumulated within the tumor microenvironment induces lactylation in immune cells, contributing to the reshaping of an immunosuppressive microenvironment and promoting tumor immune evasion (15). Furthermore, lactylation is implicated in tumor resistance to chemotherapy, targeted therapy, and immunotherapy, positioning it as a promising therapeutic target for improving treatment efficacy (16).

This article systematically reviews the molecular regulatory mechanisms of lactylation, elaborates on its functional roles in different tumor types, analyzes its associations with the tumor microenvironment and therapeutic resistance, and discusses therapeutic strategies targeting lactylation along with future research prospects. The aim is to provide new theoretical foundations and research directions for both fundamental cancer research and clinical treatment.

## 2 Molecular mechanisms and regulatory networks of lactylation

**Figure 1:**
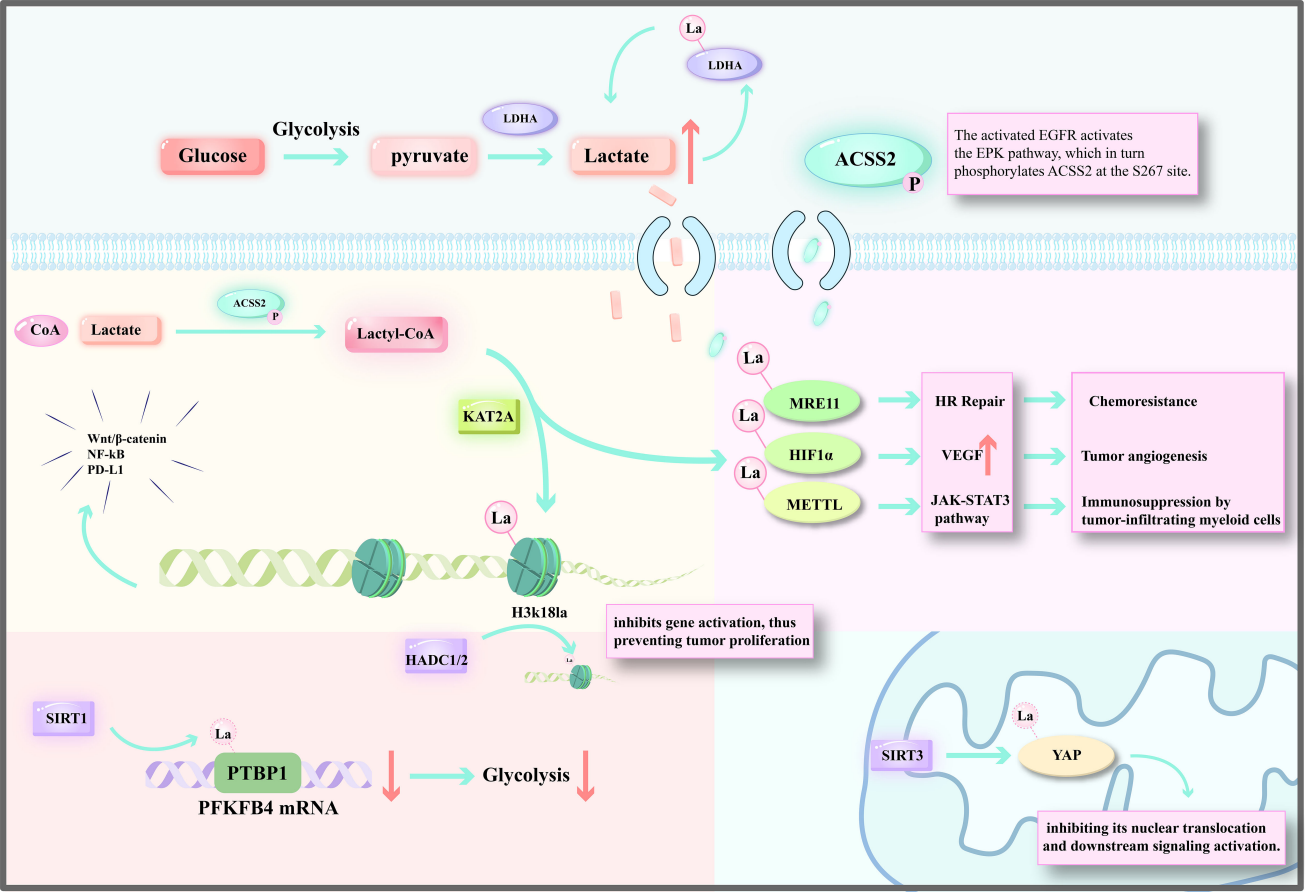
The lactate-driven lactylation modification network in tumors. EGFR activation triggers the ERK pathway, which phosphorylates ACSS2, promoting its nuclear translocation and complex formation with KAT2A. This complex converts lactate into lactyl groups to modify both histones and non-histone proteins. This process activates pro-tumorigenic genes, enhances DNA repair and immunosuppressive pathways, thereby mediating chemoresistance and immune evasion. Conversely, delactylases exert anti-tumor effects by removing these modifications, suppressing glycolysis and oncogenic signaling.

Lactylation is a post-translational modification (PTM) process characterized by the covalent attachment of lactyl groups derived from lactic acid to lysine residues on proteins. Its core significance lies in directly linking cellular metabolic status to gene expression or protein function (17). This process is governed by a sophisticated regulatory network comprising “writers” (enzymes catalyzing lactylation), “erasers” (enzymes removing lactylation), and “readers” (proteins recognizing lactylated residues), and exhibits distinct functional roles depending on whether it modifies histones or non-histone proteins (18–20) (**Figure 1**).

### 2.1 Catalytic and reverse mechanisms of lactylation

The initiation of lactylation relies on lactyl-CoA as the essential donor molecule. The synthesis of lactyl-CoA is primarily mediated by acetyl-CoA synthetase 2 (ACSS2) (2). Research has revealed that activation of the epidermal growth factor receptor (EGFR) triggers the ERK signaling pathway, which phosphorylates ACSS2 at the S267 residue. This phosphorylation promotes the nuclear translocation of ACSS2 and its formation of a complex with the lysine acetyltransferase KAT2A (2). Within this complex, ACSS2 functions as a lactyl-CoA synthetase, converting lactic acid into lactyl-CoA. Concurrently, KAT2A acts as a lactyltransferase, catalyzing lactylation of histone H3. This modification activates oncogenic pathways, including Wnt/β-catenin and NF-κB, and upregulates PD-L1 expression, thereby promoting immune evasion in glioblastoma (2). These findings establish the ACSS2-KAT2A axis as a core regulator of lactylation and identify it as a key molecular target for therapeutic intervention (**Figure 1**).

The removal of lactylation (delactylation) is primarily mediated by members of the deacetylase family. For instance, SIRT1 can regulate the stability of PTBP1 (polypyrimidine tract-binding protein 1) through delactylation (21). Specifically, in glioma stem cells, SIRT1-mediated delactylation of PTBP1 disrupts PTBP1’s ability to stabilize PFKFB4 mRNA. This leads to reduced glycolysis and consequently suppresses the maintenance of stem cell characteristics (21). SIRT3 predominantly regulates delactylation of mitochondrial proteins. In liver cancer, SIRT3-mediated delactylation of YAP inhibits its nuclear translocation and activation of downstream oncogenic signaling pathways (22). Additionally, histone deacetylases HDAC1 and HDAC2 have been confirmed to function as histone delactylases (23). In pancreatic cancer, downregulation of HDAC2, acting as an “eraser” for H3K18la, results in the accumulation of H3K18la. This accumulation activates mitotic checkpoint genes such as TTK and BUB1B, ultimately promoting tumor proliferation (24).

### 2.2 Functional differences between histone and non-histone lactylation

Histone lactylation primarily regulates gene transcription by remodeling chromatin structure (25). The most extensively studied modification sites are H3K18la and H3K9la (26, 27). For example: in colorectal cancer, enrichment of H3K18la in the promoter region of the *RUBCNL* gene activates its transcription. This activation promotes autophagosome maturation and ultimately induces resistance to the therapeutic antibody bevacizumab (28, 29). In non-small cell lung cancer (NSCLC), H3K18la directly activates *POM121* expression. This activation enhances MYC nuclear translocation and its binding to the *CD274* (PD-L1) promoter, leading to high PD-L1 expression and consequently promoting immune evasion (30).

It is important to note that the functional consequences of histone lactylation exhibit cell type specificity. For instance, during the late stage of M1 macrophage polarization, H3K18la drives the expression of anti-inflammatory genes such as *Arg1*, thereby contributing to the remodeling of an immunosuppressive tumor microenvironment (31).

Non-histone lactylation, in contrast, primarily influences protein function by altering protein conformation, stability, or interactions (3, 32). Key examples include: Lactylation of LDHA at the Y239 site enhances its catalytic activity. In pancreatic cancer, this forms a “lactate production-lactylation” positive feedback loop, accelerating lactate accumulation (24). Lactylation of MRE11 at K673 promotes its DNA binding ability, thereby enhancing homologous recombination repair (HRR). This enhancement contributes to tumor resistance to chemotherapeutic agents like cisplatin (33). Lactylation stabilizes HIF1α by inhibiting its ubiquitin-mediated degradation. Consequently, under hypoxic conditions, HIF1α continuously activates angiogenesis-related genes such as *VEGF*, promoting tumor angiogenesis (34). Lactylation of METTL3 at K18 within its zinc finger domain enhances its m6A modification activity on *Jak1* mRNA. This increased methylation activates the JAK-STAT3 pathway, promoting the immunosuppressive function of tumor-infiltrating myeloid cells (35).

### 2.3 Interplay between lactylation and other post-translational modifications

The biological effects of lactylation are not isolated but often intricately intertwined with other PTMs such as acetylation, methylation, and ubiquitination (36). This interplay significantly complicates the interpretation of lactylation-specific functional outcomes, necessitating careful dissection of its unique roles.

One major challenge arises from competitive modifications occurring at shared lysine residues. For instance, enzymes like p300/CBP catalyze both lactylation and acetylation, suggesting potential competition for identical sites (37, 38). Consequently, certain observed “lactylation effects” may in fact reflect an altered balance between lactylation and acetylation rather than being solely attributable to lactylation itself.

Furthermore, the phenomenon of enzyme sharedness adds another layer of complexity. Key delactylases, including SIRT1–3, also function as deacetylases (39–41). Thus, genetic or pharmacological inhibition of these enzymes simultaneously affects both modifications, making it difficult to discern whether phenotypic changes result specifically from altered lactylation or concurrent changes in acetylation.

Finally, lactylation engages in functional crosstalk by directly influencing other PTM pathways. It can act as a metabolic sensor that modulates downstream modifications; a notable example is lactate-induced lactylation of METTL3, which enhances its m6A methylation activity (35). This illustrates how lactylation can epigenetically regulate RNA modifications, embedding it within a broader network of post-translational cross-regulation.

## 3 Functional characteristics of lactylation across tumor types

The oncogenic effect of lactylation is tumor-type specific, and its mechanism is closely related to the characteristics of the tumor microenvironment, metabolic phenotype, and genetic background (42, 43). In tumors from different tissue origins, lactylation participates in key processes of tumor occurrence and development by regulating specific target genes or signaling pathways.

### 3.1 Colorectal cancer

**Figure 2:**
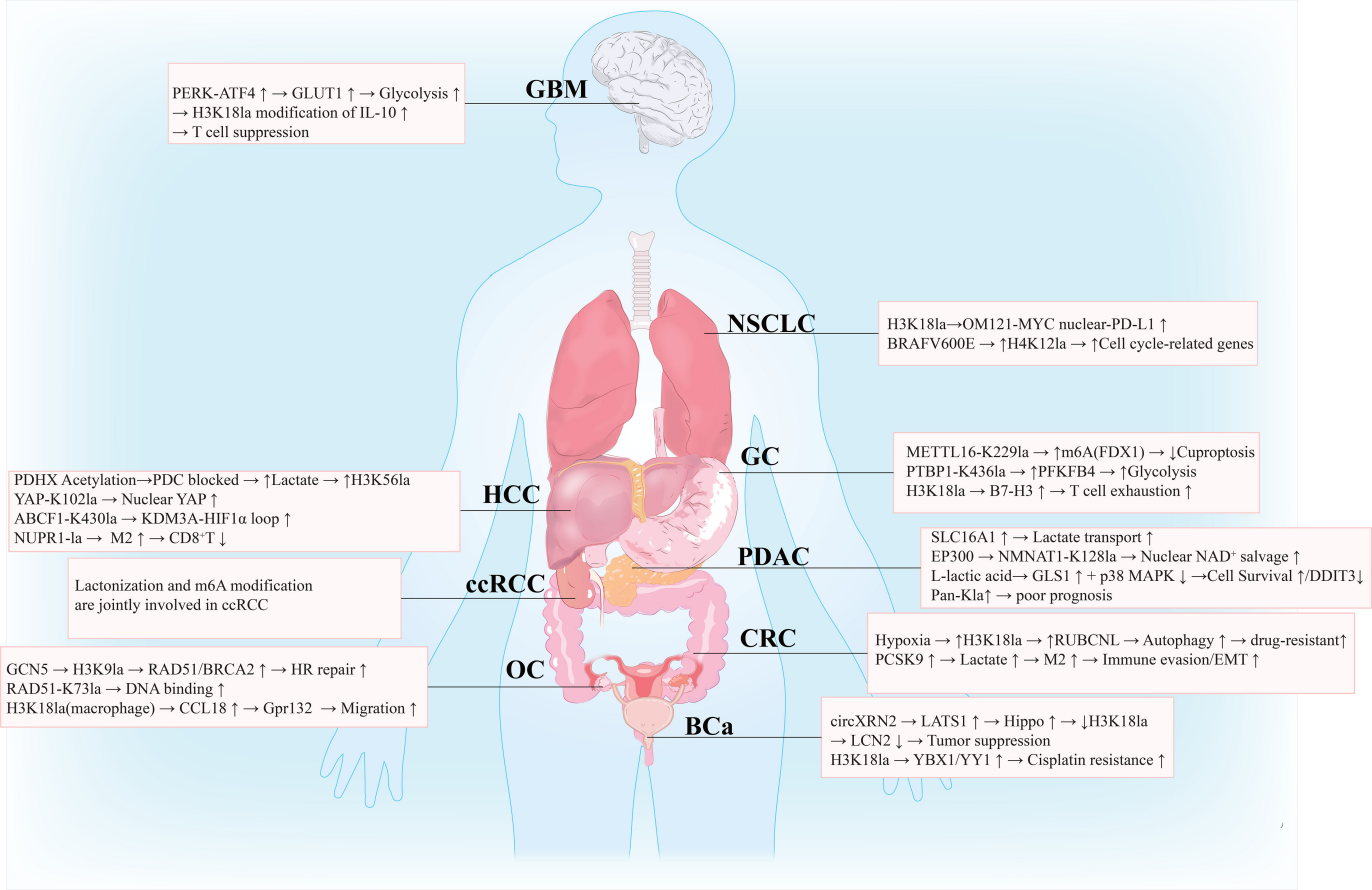
Functional mechanisms and clinical significance of lactylation in tumors. Lactylation serves as a key metabolic-epigenetic-immunological nexus, driving tumor progression by modifying histone targets (e.g., H3K18la, H3K9la) and non-histone proteins (e.g., YAP, METTL16, PDHX). Its core mechanisms include: ① Mediating mitochondrial metabolic dysfunction → lactate accumulation → epigenetic reprogramming; ② Regulating immune evasion (e.g., inducing T cell exhaustion, M2 macrophage polarization); ③ Promoting therapy resistance (e.g., chemotherapy resistance, anti-angiogenic resistance). This modification exhibits tumor-type specificity, significantly correlates with poor prognosis and reduced immunotherapy response, and targeting lactylation pathways may represent a novel therapeutic strategy.

Lactylation drives colorectal cancer (CRC) progression through multiple mechanisms (**Figure 2**). In colorectal cancer (CRC), hypoxia enhances glycolysis in CRC cells (by upregulating key enzymes such as LDHA), promoting lactate accumulation. Lactate serves as a substrate driving increased histone lactylation, particularly at H3K18 (H3K18la), a process dependent on the catalytic activity of EP300. H3K18la directly activates the transcription of the autophagy-enhancing protein RUBCNL by enriching its promoter region. Knocking down EP300 or inhibiting glycolysis significantly reduces H3K18la enrichment at the RUBCNL promoter and downregulates its expression. Transcriptionally activated RUBCNL interacts with BECN1, mediating the recruitment and functional activation of the class III phosphatidylinositol 3-kinase (PtdIns3K) complex. (28, 44). Lactylation of METTL3 in myeloid cells activates a specific signaling pathway, amplifying immunosuppressive functions and facilitating immune evasion (28). Additionally, it is linked to epithelial-mesenchymal transition (EMT), with the oncoprotein PCSK9 promoting EMT by regulating relevant molecules and signaling pathways (45).

Lactylation acts as a key metabolic link between tumor cells and the immunosuppressive microenvironment, particularly via macrophage polarization, and is regulated by PCSK9. Knockdown of PCSK9 in colon cancer cells reduces lactate levels, global protein lactylation, and macrophage migration inhibitory factor, inhibiting M2 macrophage polarization while promoting M1 polarization. This indicates that PCSK9-driven lactation and lactylation shape an immunosuppressive microenvironment favoring immune evasion and metastasis, making targeting PCSK9 or its downstream effects on lactylation a promising strategy (45).

### 3.2 Gastric cancer

The lactylation score of gastric cancer (GC) is closely related to immune cell infiltration characteristics (**Figure 2**). Lactylation of METTL16 at K229 can enhance its m6A modification of FDX1 mRNA, inhibiting cuproptosis; lactylation of PTBP1 at K436 promotes glycolysis by stabilizing PFKFB4 mRNA (41). Patients with high scores have a 2.3-fold increase in M2 macrophage infiltration in the tumor microenvironment, significantly reduced CD8^+^T cell activity, and a response rate to PD-1 inhibitors that is only 1/3 of that in patients with low scores (46). Studies have found that lactylation modification at lysine 18 of histone H3 (H3K18la), together with transcription factor Creb1 and its coactivator Ep300, binds to the promoter region of the B7-H3 gene, significantly upregulating its expression. High levels of B7-H3 suppress the proportion and cytotoxicity of tumor-infiltrating CD8^+^T cells, ultimately promoting tumor immune escape. Moreover, inhibition of lactate production reduces H3K18la modification and B7-H3 expression, restoring the anti-tumor function of CD8^+^T cells. Combining this strategy with anti-PD-1 therapy synergistically enhances tumor suppression (47).

### 3.3 Hepatocellular carcinoma

Lactylation is a critical driver of hepatocellular carcinoma progression, fundamentally linked to disrupted mitochondrial metabolism (**Figure 2**). The acetylation of PDHX (Lys 488) by p300 disrupts pyruvate dehydrogenase complex (PDC) assembly, preventing pyruvate oxidation and forcing a glycolytic shift. This metabolic reprogramming results in massive lactate production. This accumulated lactate serves as a substrate for histone lactylation, specifically at H3K56, leading to epigenetic alterations that control gene expression and facilitate tumor growth and progression (48).

Furthermore, lactylation directly modifies key oncoproteins and signaling molecules in HCC, promoting malignancy through multiple distinct pathways (49). In hepatocellular carcinoma, lactate produced by lactate dehydrogenase A (LDHA) promotes lactylation modification of Yes-associated protein (YAP) at lysine 102 (K102). This modification antagonizes phosphorylation at serine 127 (Ser127) of YAP, thereby inhibiting its phosphorylation and leading to activation. The activated YAP undergoes nuclear translocation and binds to the TEAD transcription factor to regulate the expression of downstream target genes. (50, 51). Additionally, within the tumor microenvironment, lactylation of NUPR1 in tumor-associated macrophages promotes immunosuppressive M2 polarization by inhibiting the ERK/JNK pathway, leading to reduced CD8+ T cell infiltration and immune evasion. Thus, lactylation acts as a pervasive mechanism regulating diverse oncogenic processes in HCC (52).

### 3.4 Non-small cell lung cancer

Lactylation modification significantly promotes the malignant progression of non-small cell lung cancer (NSCLC) through multiple mechanisms, and its core roles can be summarized into the following three aspects:

Firstly, lactylation drives the expression of key oncogenes through epigenetic regulation. Studies have found that histone H3 lysine 18 lactylation (H3K18la) is significantly elevated in NSCLC tissues and cells and is directly enriched in the promoter regions of oncogenes such as YTHDF2, activating their transcription (30, 53). As an m6A reader protein, YTHDF2 recognizes and stabilizes the m6A modification of downstream targets like SFRP2, further promoting tumor glycolysis and stemness (30, 53). Additionally, lactylation modification can indirectly enhance tumor cell proliferation, migration, and glycolytic metabolism by suppressing the transcription of tumor suppressor genes such as PTEN (54).

Secondly, lactylation regulates the stability and function of tumor-related proteins through non-histone modifications. For instance, lactate directly modifies lysine 850 of the RNA-binding protein RBM15, inhibiting its ubiquitination-mediated degradation and enhancing its binding ability to the METTL3 complex (55). This leads to a global increase in m6A methylation levels, promoting tumor proliferation and migration. Similarly, lactylation of the transcription factor SOX9 is enhanced under hypoxic conditions, thereby promoting tumor stemness, invasion, and metastatic capabilities (56).

Lastly, lactylation also plays a critical role in immune evasion and targeted therapy resistance in NSCLC. H3K18la can activate the transcription of POM121, promote MYC nuclear translocation, and facilitate its binding to the CD274 (PD-L1) promoter, leading to upregulated PD-L1 expression and subsequent suppression of CD8+ T cell cytotoxicity (30). In EGFR-TKI resistance models, NNMT depletes the methyl donor, suppressing repressive histone methylations (H3K9me3 and H3K27me3), while ALDH3A1 activation increases lactate production (57). This lactate further upregulates NNMT expression via p300-mediated H3K18la, forming a dual positive feedback loop (EGR1/NNMT/EGR1 and NNMT/ALDH3A1/lactate/NNMT), ultimately resulting in the development of drug resistance (57) (**Figure 2**).

### 3.5 Glioblastoma

Lactylation modification drives malignant progression in glioblastoma (GBM) through multiple mechanisms, with its core roles primarily manifested in reprogramming the immune microenvironment and mediating therapy resistance. In terms of immune regulation, high glycolytic activity in tumor cells and tumor-associated macrophages (such as monocyte-derived macrophages, MDMs) leads to lactate accumulation, which induces histone lactylation (e.g., H3K9la, H3K18la) and subsequently activates the transcription of immunosuppressive genes (58). For instance, lactate-driven histone lactylation in MDMs promotes IL-10 expression and suppresses T cell function (58). Meanwhile, lactate produced by the microenvironment and glioma stem cells (GSCs) enhances histone lactylation through the CBX3-EP300 complex, upregulates CD47 expression, inhibits macrophage phagocytosis, and promotes immune evasion (59).

In terms of therapy resistance, lactylation modification contributes to radiation and temozolomide (TMZ) resistance by affecting DNA repair, stem cell properties, and epigenetic regulation (60). For example, in ALDH1A3hi GBM, PKM2 tetramerization promotes lactate accumulation, leading to lactylation of the XRCC1 protein (at K247), enhancing its nuclear translocation and DNA repair capacity (61). Histone H3K9 lactylation activates LUC7L2 transcription, causing intron retention in MLH1 and impairing mismatch repair function, thereby mediating TMZ resistance (62). Additionally, the transcription of USP4 is regulated by H3K18 lactylation; it stabilizes ANXA2 through deubiquitination, activates the STAT3 pathway, and maintains stemness and radioresistance in glioblastoma stem cells (63).

In summary, the core role of lactylation modification in GBM lies in tightly linking high glycolytic metabolism with immunosuppression and therapy resistance through epigenetic mechanisms, providing a theoretical basis for combined therapeutic strategies targeting both metabolism and epigenetics.

### 3.6 Bladder cancer

In bladder cancer (BCa), circXRN2 exerts a tumor-suppressive effect by inhibiting H3K18la (**Figure 2**). circXRN2 can bind to the SPOP degradation domain of LATS1, preventing its ubiquitin-mediated degradation, thereby activating the Hippo pathway, downregulating H3K18la and downstream LCN2 expression (64). In terms of treatment, H3K18la can promote cisplatin resistance by activating YBX1/YY1 transcription factors, and targeted inhibition of H3K18la can restore cisplatin sensitivity in resistant cells by 3.5 times (65). Additionally, Lactylation modification in bladder cancer promotes the polarization of M2 tumor-associated macrophages by regulating epigenetic mechanisms, thereby driving immunosuppression. Specifically, histone H3K18 lactylation (H3K18la) has been demonstrated to directly enhance the expression of the PRKN gene. PRKN-mediated mitophagy is a critical process for M2 macrophage polarization, and its activation leads to the establishment of an immunosuppressive phenotype, ultimately promoting immune evasion and tumor progression in bladder cancer (66).

### 3.7 Ovarian cancer

Lactylation plays a central role in ovarian cancer by regulating gene expression and the tumor microenvironment, thereby driving chemotherapy resistance, immune evasion, and tumor progression. Its mechanism of action is primarily twofold: Firstly, lactate induces lactylation of histones (e.g., H3K18la, H3K9la, H4K12la) and key DNA repair proteins (e.g., RAD51), which directly activates the expression of genes involved in homologous recombination repair (HRR) (such as RAD51, BRCA2, RUBCNL) or drug resistance genes (e.g., RAD23A) (67–69). This enhances the DNA damage repair capacity, leading to resistance to platinum-based drugs or niraparib (69). Secondly, lactylation promotes immune escape by modulating the expression of immune-related genes. For instance, in tumor cells, LDHB-mediated H3K18la upregulates PD-L1 expression (70). In tumor-associated macrophages, lactate activates CCL18 expression via H3K18la, inducing M2 polarization and suppressing T cell function, thus fostering an immunosuppressive microenvironment (71) (**Figure 2**).

The upstream drivers of lactylation primarily stem from enhanced glycolysis or glutamine metabolism. Chemotherapy stress or a hypoxic microenvironment can promote lactate production from glutamine via ACAT1-mediated acetylation of ME2, or amplify glycolytic flux through lactylation of glycolytic enzymes like PFKP (e.g., at the K392 site), creating a positive feedback loop (72). Therefore, targeting key regulators of lactylation (e.g., ACAT1, GCN5, LDHB, or PFKP) or downstream effector genes (e.g., RAD23A, CCL18) could effectively reverse drug resistance and immunosuppression, offering novel combination strategies for ovarian cancer treatment (67, 70, 72, 73).

In summary, lactylation constitutes a central bridge connecting metabolic reprogramming, DNA repair, the immune microenvironment, and gene transcription in ovarian cancer, serving as a critical driver of its malignant progression and therapy resistance.

### 3.8 Renal cell carcinoma

Lactylation serves as a central mechanism driving renal cell carcinoma (RCC) progression through its dual role in metabolic reprogramming and epigenetic regulation. Within the hypoxic, lactate-rich tumor microenvironment, lactylation modifies key metabolic enzymes—such as mitochondrial MDH2 and glycolytic LDHA—enhancing tricarboxylic acid cycle efficiency, promoting NADPH-dependent ROS clearance, and reinforcing the Warburg effect. This metabolic rewiring equips tumor cells with heightened adaptability and antioxidant capacity (74) (**Figure 2**).

Furthermore, lactate accumulation induces lactylation on histones and RNA-binding proteins like YTHDC1, stabilizing pro-tumor mRNAs such as BCL2 and E2F2 (75). Notably, FKBP10 promotes LDHA phosphorylation and lactate production, which in turn stimulates histone lactylation, forming a feedforward loop that augments malignant progression and modulates response to HIF2α blockade therapy (76). Additionally, inactive VHL triggers histone lactylation that activates PDGFRβ signaling, which further amplifies lactylation, creating a pathogenic positive feedback cycle (77). Together, these findings underscore lactylation as a critical integrative node in RCC pathogenesis and highlight its potential as a target for prognostic and therapeutic strategies.

### 3.9 Pancreatic adenocarcinoma

Lactylation, particularly lysine lactylation, plays a pivotal role in pancreatic adenocarcinoma (PDAC) progression (**Figure 2**). A novel lactylation-related prognostic signature based on five genes (SLC16A1, HLA-DRB1, KCNN4, KIF23, and HPDL) was constructed, which effectively predicts overall survival, immune status, and treatment response in PDAC patients (78). Among these, SLC16A1 was identified as a key regulator: it modulates lactylation through lactate transport, and its downregulation, along with reduced lactylation, significantly inhibits tumor progression both *in vitro* and *in vivo*, highlighting its potential as a therapeutic target (78).

Mechanistically, l-lactate promotes PDAC cell survival under glucose deprivation by supporting mitochondrial respiration via GLS1-mediated glutaminolysis. It enhances the NMNAT1-mediated NAD+ salvage pathway while inactivating p38 MAPK signaling and suppressing DDIT3 transcription. Notably, pan-lysine lactylation (Pan-Kla) is upregulated in PDAC and linked to poor prognosis; EP300 catalyzes lactylation of NMNAT1 at Lys128, enhancing its nuclear localization and enzymatic activity to sustain nuclear NAD+ salvage, thereby facilitating cancer growth. This unravels l-lactate’s role in supporting PDAC survival and offers new therapeutic directions targeting lactylation and NAD+ metabolism (79).

### 3.10 Breast cancer

Lactylation plays a pivotal role in breast cancer progression by linking metabolic reprogramming to epigenetic regulation and gene expression. In breast cancer, elevated glycolytic activity leads to lactate accumulation, which drives protein lactylation—particularly on histones—altering transcriptional programs that promote malignancy. For instance, KCNK1 upregulation activates LDHA, increasing lactate production and enhancing H3K18 lactylation, which in turn stimulates expression of pro-tumorigenic genes and reinforces glycolytic flux via a positive feedback loop (80). Similarly, H4K12 lactylation in triple-negative breast cancer (TNBC) suppresses Schlafen 5 (SLFN5), reducing apoptosis and accelerating disease progression (81).

Mechanistically, lactylation modifies both histone and non-histone proteins to regulate key oncogenic pathways. Lactate-induced histone lactylation at gene promoters—such as that of c-Myc—enhances its expression, subsequently activating splicing factor SRSF10 to promote alternative splicing of MDM4 and Bcl-x, thereby supporting proliferation and survival (82). Non-histone lactylation is also significant: high glucose conditions induce RCC2 lactylation at K124 via KAT2A, stabilizing MAD2L1 mRNA and driving cell cycle progression (83). Furthermore, lactylation modulates therapy resistance; in TNBC, cancer-associated fibroblasts (CAFs) enhance ZFP64 expression through histone lactylation, inhibiting ferroptosis and conferring doxorubicin resistance (84).

These findings underscore the clinical relevance of lactylation in breast cancer, highlighting its role in tumor growth, metastasis, and treatment resistance. Targeting lactylation-related mechanisms—such as LDHA activity, lactylation writers or erasers like KAT2A or HDACs, or downstream effectors like ZFP64—may offer novel therapeutic strategies for breast cancer, particularly in aggressive subtypes such as TNBC where metabolic-epigenetic crosstalk is pronounced.

## 4 Cross-regulation between lactylation and the tumor microenvironment

**Figure 3:**
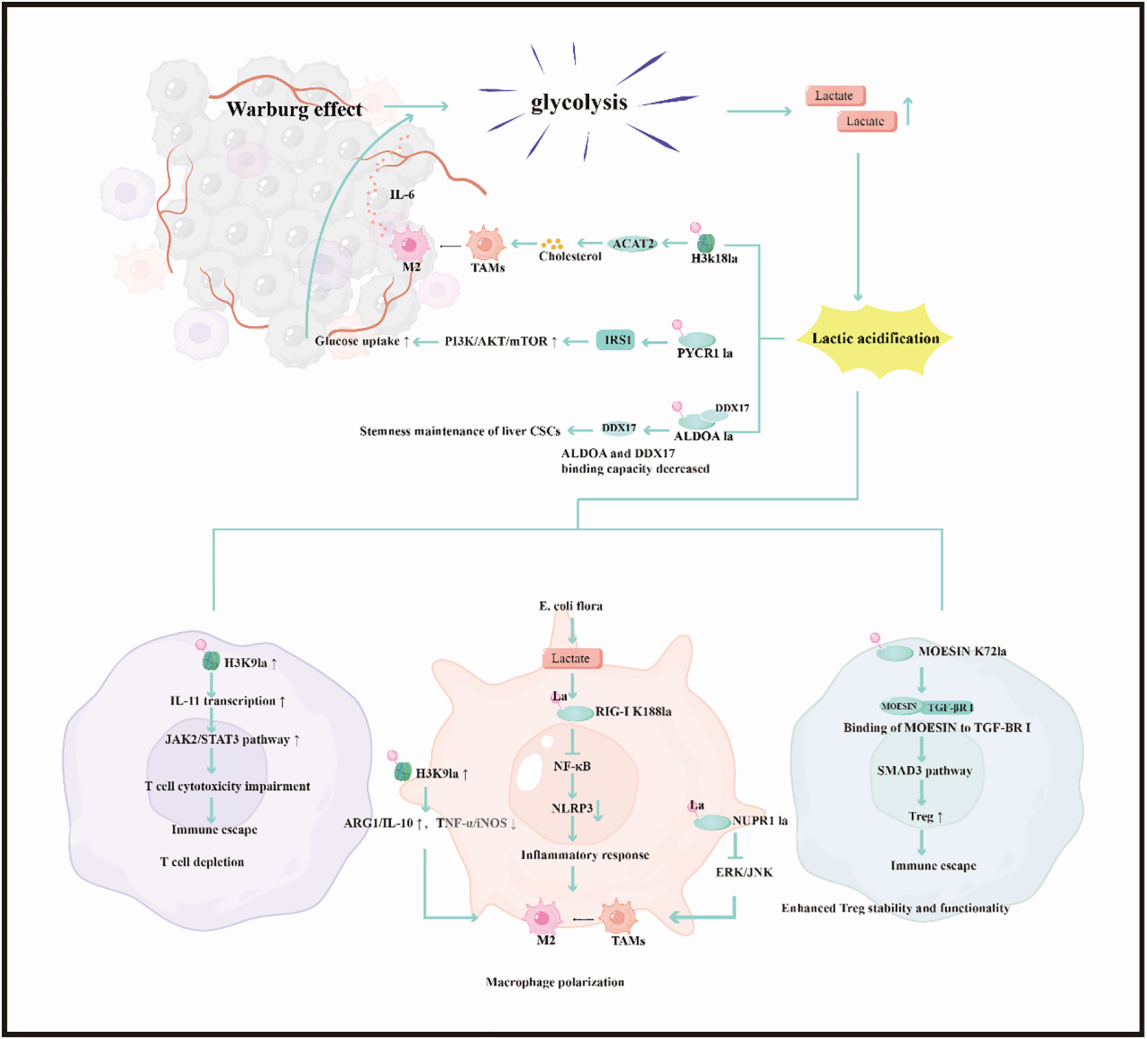
Cross-regulation between lactylation and the tumor microenvironment. Tumor metabolic reprogramming (Warburg effect) drives lactate accumulation, promoting lactylation modifications (e.g., H3K18la). Lactylation enhances cholesterol synthesis, the PI3K/AKT/mTOR pathway, and liver CSC stemness maintenance through epigenetic regulation (e.g., activating ACAT2, PYCR1). Concurrently, lactylation modifications (e.g., MOESIN K72la, RIG-I K188la) remodel the immune microenvironment, inducing M2 polarization of TAMs, enhanced Treg functionality, and T cell exhaustion. This establishes a “lactylation-metabolism-immunosuppression” feedforward loop, promoting tumor immune escape.

The unique metabolic landscape of the tumor microenvironment (TME) creates ideal conditions for lactylation to occur. In turn, lactylation actively reshapes the TME to promote tumor progression, establishing a self-perpetuating vicious cycle of metabolic-epigenetic regulation (11). This intricate cross-talk manifests primarily in two interconnected facets: the reciprocal relationship between metabolic reprogramming and lactylation, and the profound influence of lactylation on immune cell function within the TME (11) (**Figure 3**).

### 4.1 Metabolic reprogramming fuels and is modulated by lactylation

The Warburg effect, a hallmark of tumor metabolism, leads to profound lactate accumulation within the TME, providing abundant substrate for lactylation (85, 86). Evidence supporting this causal link comes from experiments showing that genetic knockout of lactate dehydrogenase A/B (LDHA/B), which blocks glycolytic flux to L-lactate under hypoxia, completely abolished the induction of specific lactylation marks (e.g., KL-la) while increasing others (KD-la, Kce) (87). This high lactate environment can induce LDHA expression through HIF1α-dependent mechanisms, further enhancing lactate production (88).

### 4.2 Lactylation can reversely regulate metabolic pathways, forming feedback regulation

In pancreatic cancer (PDAC), H3K18la activates *ACAT2* transcription, boosting cholesterol synthesis. Tumor-derived cholesterol is then transferred to tumor-associated macrophages (TAMs) via small extracellular vesicles (sEVs), reinforcing their immunosuppressive M2 polarization. In a reciprocal loop, IL-6 secreted by these M2 TAMs further stimulates glycolysis in tumor cells, completing a “lactylation-cholesterol-immunosuppression” feedback circuit (89, 90). Lactylated PYCR1 can activate *IRS1* expression via H3K18la modification. This enhances the PI3K/AKT/mTOR signaling pathway, driving increased glucose uptake by tumor cells and further fueling glycolysis and lactate production (91, 92). In liver cancer, lactylation of ALDOA at residues K230 and K322 weakens its binding to DDX17. This unleashes DDX17’s regulatory function on stemness-related genes, thereby helping to maintain the stem-like properties of liver cancer stem cells (LCSCs) (93) (**Figure 3**).

## 5 Regulation of immune cell function by lactylation

### 5.1 Lactylation induces functional exhaustion in T cells

In head and neck squamous cell carcinoma (HNSCC), the high-lactate tumor microenvironment elevates H3K9la levels in CD8^+^ T cells, which activates IL-11 transcription and initiates the JAK2/STAT3 pathway (94). Mechanistically, overexpression of IL-11 promotes tumor progression *in vivo* while impairing CD8^+^ T cell function, thereby reducing their tumor-killing capacity (94). Clinically, H3K9la levels positively correlate with IL-11 expression and predict poor responses to immunotherapy in HNSCC patients. This study highlights the critical role of histone lactylation in mediating immune escape, offering valuable insights for developing improved immunotherapeutic strategies against HNSCC (94, 95) (**Figure 3**).

### 5.2 Lactylation phenotype determines macrophage functional polarization

In M2-type tumor-associated macrophages (TAMs), H3K18la activates transcription of anti-inflammatory genes such as ARG1 and IL-10, while suppressing M1-type markers including TNF-α and iNOS (96). In glioblastoma (GBM), tumor cell-derived lactate induces lactylation of RIG-I at K188 in macrophages, which inhibits NF-κB recruitment to the Nlrp3 promoter and reduces inflammasome activation (97). Additionally, lactylated NUPR1 maintains the M2 phenotype of TAMs by inhibiting the ERK/JNK pathway (52). In a colorectal cancer (CRC) liver metastasis model, tumor-resident Escherichia coli enhances lactate production, which further inhibits Nlrp3 transcription through RIG-I lactylation, promoting M2 polarization and the formation of an immunosuppressive microenvironment (97) (**Figure 3**).

### 5.3 Lactylation regulates the stability of regulatory T cells

In liver cancer, lactate induces lactylation of MOESIN at K72, enhancing its interaction with TGF-β receptor I and activating the SMAD3 pathway to promote Treg differentiation and function (98). Hepatocellular carcinoma patients responding to anti-PD-1 therapy exhibit lower MOESIN lactylation levels in Tregs compared to non-responders (98). Collectively, lactate and its derivative lactylation play pivotal roles in modulating immune responses within the tumor microenvironment (TME), particularly affecting T-cell-mediated cancer immunotherapy. Elevated lactate levels, a hallmark of the Warburg effect, contribute to immune suppression by impairing CD8^+^ T cell functionality and promoting regulatory T cell (Treg) activity (99) (**Figure 3**).

## 6 Association mechanisms between lactylation and tumor therapeutic resistance

Lactylation contributes to tumor resistance to chemotherapy, targeted therapy, and immunotherapy through multiple pathways, emerging as a key factor in treatment failure (65, 100–104). In-depth exploration of its molecular mechanisms may provide novel intervention strategies to reverse drug resistance (105).

### 6.1 Chemotherapy resistance

Chemoresistance remains a critical challenge in cancer treatment, and lactylation has been identified as a pivotal regulator in this process, with distinct mechanisms across different chemotherapeutic agents and cancer types.

**Figure 4:**
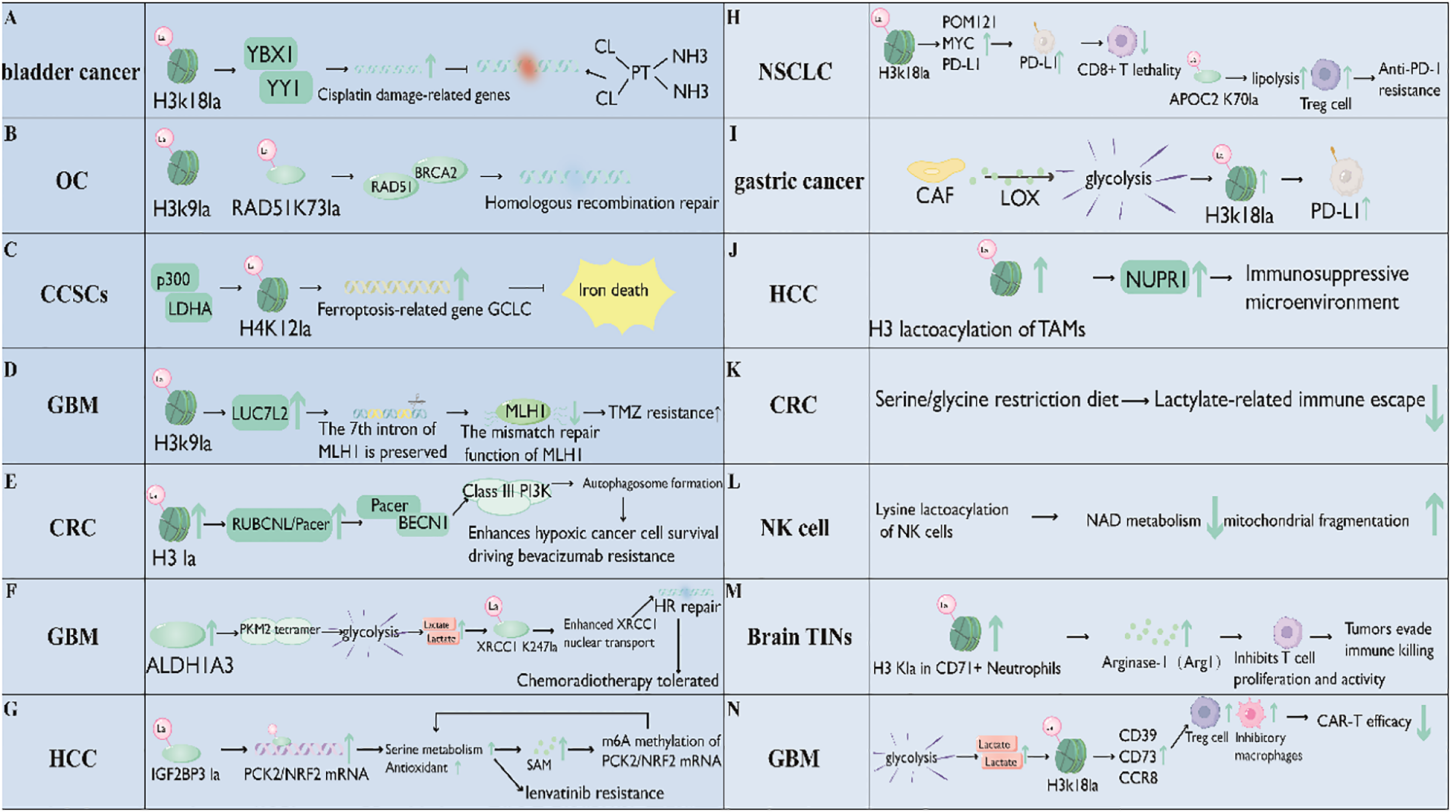
Lactylation drives tumor resistance to chemotherapy, targeted therapy, and immunotherapy. Key mechanisms include: Chemoresistance: Histone (e.g., H3K18la, H4K12la) or non-histone (e.g., RAD51K73la, XRCC1K247la) modifications enhance DNA repair (e.g., HR, MMR) or inhibit ferroptosis; Targeted therapy resistance: Lactate accumulation induces lactylation (e.g., IGF2BP3, RUBCNL), activating metabolic reprogramming (serine metabolism) or autophagy to promote survival; Immunotherapy resistance: Lactate-driven histone (H3K18la) or non-histone (APOC2-K70la) modifications upregulate PD-L1, suppress T/NK cells, and induce an immunosuppressive microenvironment. Targeting lactylation or lactate metabolism reverses resistance.

Cisplatin resistance, often linked to enhanced DNA repair capacity, is modulated by lactylation in various cancers (65). In bladder cancer, H3K18 lactylation (H3K18la) activates the transcription of YBX1 and YY1, which form a complex and bind to the promoter regions of cisplatin damage-related genes, thereby promoting their expression (65) (**Figure 4**). Notably, H3K18la plays a crucial role in activating target gene transcription by accumulating in their promoter regions. Targeted inhibition of H3K18la effectively restores cisplatin sensitivity in cisplatin-resistant epithelial cells (52).

In ovarian cancer (OC), lactylation is closely associated with platinum resistance, a major obstacle in treatment. One study reveals that elevated histone lactylation (particularly H3K9la) and non-histone RAD51K73la in platinum-resistant ovarian cancer enhance homologous recombination (HR) repair by activating the expression of RAD51 and BRCA2, thereby conferring cisplatin resistance. Both modifications share the upstream regulator GCN5, and a GCN5 inhibitor improves cisplatin efficacy *in vivo* (67) (**Figure 4**). Another study identifies 14 lactylation-related genes in ovarian cancer, showing that the high-risk group exhibits higher half-maximal inhibitory concentration (IC50) values for cisplatin compared to the low-risk group, with differential gene expression linked to pathways involved in cancer progression and drug response. Collectively, these findings indicate that lactylation contributes to chemoresistance in ovarian cancer through mechanisms such as regulating HR repair and forming specific gene signatures, offering potential therapeutic targets and prognostic indicators (23).

Chemoresistance in colorectal cancer stem cells (CCSCs) is closely linked to histone lactylation. Specifically, histone H4 lysine 12 lactylation (H4K12la) enhances CCSC chemoresistance, with p300 catalyzing this lactylation and HDAC1 mediating its delactylation (106). H4K12la upregulates the expression of the ferroptosis-related gene GCLC, thereby inhibiting ferroptosis and contributing to chemoresistance in CCSCs. Notably, inhibiting p300 or LDHA reduces H4K12la levels, increasing CCSC sensitivity to chemotherapy. Additionally, the GCLC inhibitor BSO promotes ferroptosis and sensitizes CCSCs to oxaliplatin, suggesting that targeting p300, LDHA, or GCLC may be a potential strategy to overcome tumor chemoresistance (106) (**Figure 4**).

Lactylation is also closely associated with temozolomide (TMZ) resistance in glioblastoma (GBM) (62). Specifically, histone H3 lysine 9 lactylation (H3K9la) induces TMZ resistance in GBM through a mechanism involving LUC7L2-mediated intron 7 retention of MLH1. Lactylation levels are upregulated in recurrent GBM tissues and TMZ-resistant cells, with H3K9la as the primary form, which significantly accumulates in the LUC7L2 promoter to activate its transcription and promote LUC7L2 expression (62). LUC7L2 then mediates intron 7 retention of MLH1, reducing MLH1 expression and inhibiting mismatch repair (MMR), ultimately leading to TMZ resistance. Importantly, the anti-epileptic drug stiripentol, which inhibits lactate dehydrogenase A/B (LDHA/B) activity and acts as a lactylation inhibitor, enhances the sensitivity of GBM cells to TMZ both *in vitro* and *in vivo*. This highlights the role of lactylation in TMZ resistance in GBM and its potential as a therapeutic target to overcome such resistance (62) (**Figure 4**).

### 6.2 Targeted therapy resistance

In metastatic colorectal cancer (CRC), histone lactylation is closely linked to resistance to the targeted therapy bevacizumab. CRC patients resistant to bevacizumab exhibit elevated histone lactylation levels, driven by lactate accumulation in the tumor microenvironment, which is further induced by enhanced cellular glycolysis under hypoxic conditions. Mechanistically, histone lactylation promotes the transcription of RUBCNL/Pacer, which facilitates autophagosome maturation by interacting with BECN1 and mediating the recruitment and function of the class III phosphatidylinositol 3-kinase complex. This supports the proliferation and survival of hypoxic cancer cells, contributing to bevacizumab resistance (28) (**Figure 4**).

In ALDH1A3-high GBM, lactate accumulation—driven by enhanced PKM2 tetramerization via ALDH1A3-PKM2 interaction—induces lactylation of XRCC1 at K247. This modification strengthens XRCC1’s affinity for importin α, promotes its nuclear translocation, and enhances DNA repair, thereby mediating chemoradiotherapy resistance. Targeting the ALDH1A3-PKM2 interaction disrupts this lactate-related resistance mechanism, improving responses to chemoradiotherapy (61) (**Figure 4**).

In advanced hepatocellular carcinoma (HCC), acquired resistance to the molecular-targeted therapy lenvatinib is associated with lysine lactylation of IGF2BP3, which arises from increased glycolysis and subsequent lactate accumulation in lenvatinib-resistant models (107). This lactylation of IGF2BP3 is critical for capturing PCK2 and NRF2 mRNAs, enhancing their expression to reprogram serine metabolism and strengthen the antioxidant defense system, thereby promoting lenvatinib resistance. Furthermore, altered serine metabolism increases methylated substrates such as S-adenosylmethionine (SAM), facilitating N6-methyladenosine (m6A) methylation of PCK2 and NRF2 mRNAs. This forms a regulatory loop that maintains their elevated expression and sustains resistance (107) (**Figure 4**).

### 6.3 Immunotherapy resistance

Lactylation, driven by lactate accumulation often linked to enhanced glycolysis, plays a critical role in mediating resistance to immunotherapy across various cancers by promoting immune evasion. In non-small cell lung cancer (NSCLC), histone H3 lysine 18 lactylation (H3K18la) activates the POM121/MYC/PD-L1 pathway, upregulating PD-L1 and impairing CD8+ T-cell cytotoxicity. Meanwhile, non-histone lactylation of APOC2 at K70 stabilizes the protein, triggering lipolysis, regulatory T-cell accumulation, and resistance to anti-PD-1 therapy (30). Similarly, in gastric cancer, cancer-associated fibroblasts (CAFs) secrete LOX to induce glycolysis and lactate buildup, leading to H3K18la-mediated PD-L1 transcription, which reduces the efficacy of PD-1/PD-L1 blockade (108). In hepatocellular carcinoma (HCC), tumor-derived lactate promotes histone lactylation in tumor-associated macrophages, upregulating NUPR1 and creating an immunosuppressive microenvironment that impairs immunotherapy responses (52, 109) (**Figure 4**).

Targeting lactylation, either directly or through metabolic interventions, emerges as a promising strategy to sensitize tumors to immunotherapy. For example, antibodies against lactylated APOC2-K70 enhance anti-PD-1 efficacy in NSCLC models. Inhibiting glycolysis reduces lactate levels and lactylation, restores CD8+ T-cell function, and improves immunotherapy responses in NSCLC and gastric cancer (30, 108). In colorectal cancer, a serine/glycine-free diet reduces lactylation-related immune evasion, promotes cytotoxic T-cell accumulation, and synergizes with PD-1 inhibitors to enhance antitumor immunity (110) (**Figure 4**).

Tumor-derived lactate in the tumor microenvironment induces lysine lactylation (Kla) in natural killer (NK) cells, leading to impaired NAD metabolism, mitochondrial fragmentation, and reduced cytotoxicity, thereby contributing to immunotherapy resistance. Targeting Kla restores NK cell function by modulating Kla on ROCK1, inhibiting ROCK1-DRP1 signaling to prevent mitochondrial fragmentation, and thus enhancing the efficacy of NK cell-based immunotherapy against lactate-induced resistance (111) (**Figure 4**).

In brain tumor-infiltrating neutrophils, hypoxia drives enhanced glucose metabolism in the CD71^+^ subset, leading to high lactate production. This lactate induces histone lactylation, which regulates arginase-1 expression and endows CD71^+^ neutrophils with immunosuppressive properties. Targeting histone lactylation blocks the immunosuppressive ability of these CD71^+^ neutrophils (112) (**Figure 4**).

In the context of CAR-T immunotherapy for solid tumors such as glioblastoma, lactate produced by tumor glycolysis contributes to an immunosuppressive microenvironment and impairs CAR-T efficacy, with mechanisms linked to histone lactylation. Specifically, intracellular lactate accumulation upregulates the expression of CD39, CD73, and CCR8 in CD4^+^ T cells and macrophages by enhancing the activity of their gene promoters through histone H3K18 lactylation, promoting regulatory T-cell infiltration and CAR-T immunosuppression. Importantly, inhibiting lactate generation alleviates this immunosuppression, reduces tumor-infiltrating CAR-Treg cells, and enhances CAR-T immune activation. This indicates that targeting lactate-driven lactylation is a potential strategy to improve CAR-T immunotherapy efficacy in solid tumors (113) (**Figure 4**).

These findings underscore a strong link between lactylation and immunotherapy resistance, where lactate-driven lactylation of histones and non-histone proteins modulates immune checkpoint molecules and tumor microenvironment dynamics. Combining lactylation-targeted strategies with immune checkpoint inhibitors holds significant potential to overcome resistance and improve immunotherapeutic outcomes across multiple cancer types.

## 7 Therapeutic strategies and research progress targeting lactylation

Therapeutic strategies targeting lactylation have emerged as a new focus in cancer research, spanning multiple fields such as metabolic intervention, epigenetic regulation, and combined immunotherapy. Some of these strategies have shown promising prospects in preclinical models.

### 7.1 Metabolic pathway intervention

Interventions targeting lactate metabolism hold promise for modulating immune sensing and inhibiting tumor growth. For example, inhibiting lactate production or lactate-mediated lactylation can disrupt the degradation of cGAS—a key sensor of cytosolic DNA—thereby restoring interferon production and suppressing tumor progression. Such interventions may target specific processes, including the lactylation of cGAS at K21 or the phosphorylation of PSMA4 (114, 115).

In prostate cancer, metabolic pathway intervention can counteract angiogenesis and vasculogenic mimicry. Inhibiting MCT1, a transporter that imports lactate into tumor cells, reduces lactate-induced stabilization of HIF1α via lactylation, thereby downregulating KIAA1199 and its pro-angiogenic effects. Additionally, the hyaluronic acid (HA) biosynthesis inhibitor 4MU reverses KIAA1199-driven angiogenesis, highlighting metabolic targeting as a viable strategy to hinder prostate cancer progression (34).

**Figure 5:**
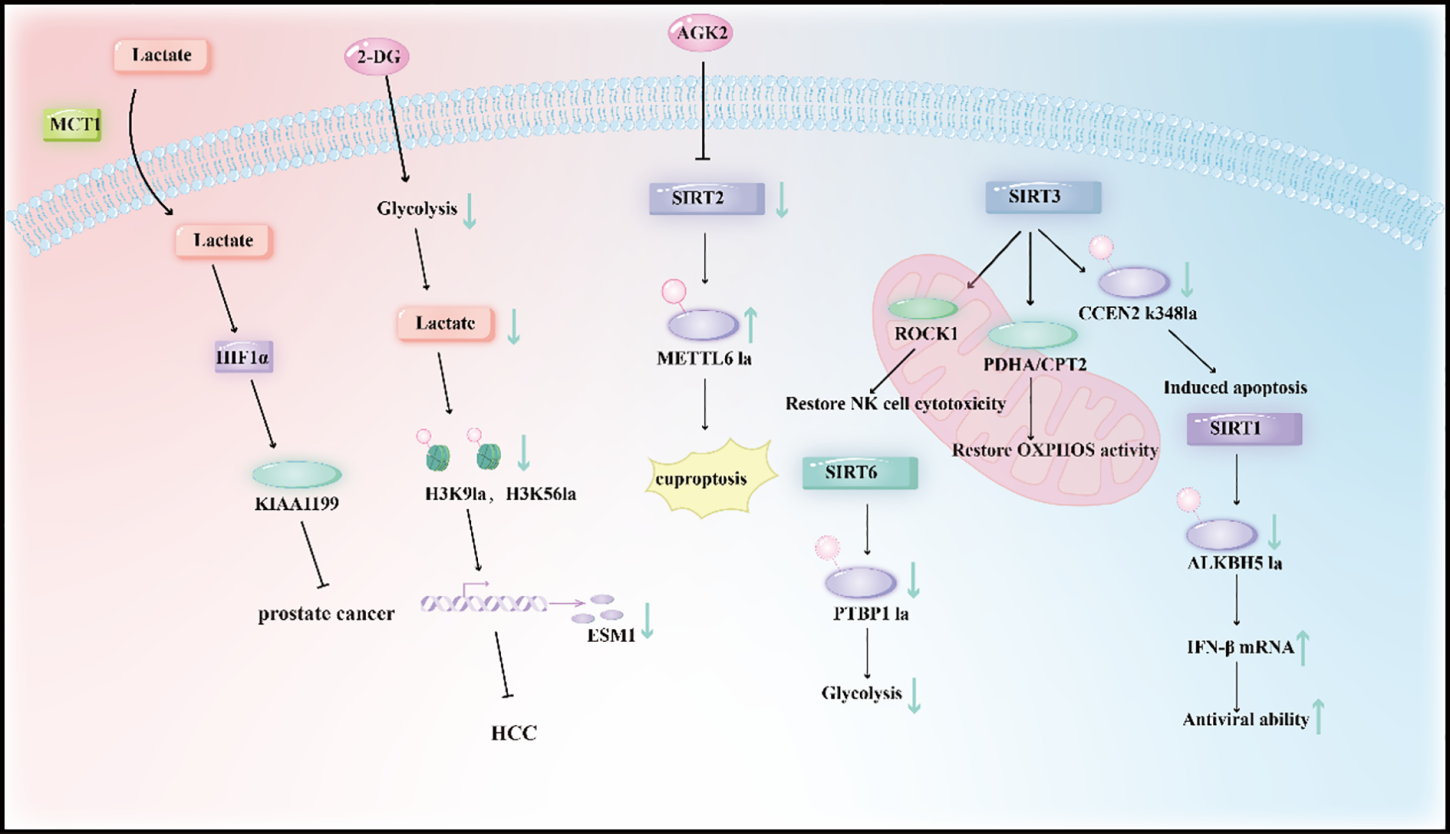
Therapeutic strategies targeting lactylation focus on metabolic intervention and epigenetic regulation. Metabolic Intervention: 2-DG inhibits glycolysis to reduce lactate accumulation, downregulates histone lactylation (e.g., H3K9la/H3K56la), and blocks ESM1’s prooncogenic effects (in HCC); MCT1 inhibitors impede lactate uptake, suppressing HIF1α lactylation-mediated KIAA1199-driven angiogenesis (in prostate cancer).Epigenetic Regulation: The SIRT family (SIRT1/2/3/6) acts as core delactylases, reversing lactylation modifications of METTL16 (cuproptosis), PTBP1 (glycolysis), CCNE2 (apoptosis), and ROCK1 (NK cell cytotoxicity) to inhibit tumor progression.

In hepatocellular carcinoma (HCC), metabolic intervention through glycolysis inhibition effectively suppresses malignant phenotypes. 2-DG reduces lactate accumulation and subsequent histone lactylation, which in turn downregulates the transcription of ESM1—a key driver of cell proliferation, migration, and metastasis. This intervention not only impairs tumor growth and metastasis *in vitro* but also reduces *in vivo* tumor progression, underscoring its therapeutic potential in HCC (116) (**Figure 5**).

### 7.2 Epigenetic regulation

Epigenetic modifications, particularly histone lysine lactylation, are closely linked to lactate metabolism and cellular responses to hypoxia (117). Specifically, lysine L-lactylation (KL-la)—rather than its isomers KD-la or Kce—is the primary lactylation modification induced by hypoxia. Driven by glycolysis-derived L-lactate, KL-la is associated with the expression of hypoxia-inducible factor 1 alpha (HIF-1α), thereby mediating tumor cell adaptation to hypoxic environments. This modification is dynamically regulated, with p300/CBP identified as a key contributor to hypoxia-induced KL-la, highlighting its role as an epigenetic mediator of metabolic-epigenetic crosstalk (10, 24, 87, 118–122) (**Figure 5**).

The SIRT family of proteins, known as NAD+-dependent deacetylases, also function as key delactylases, playing a critical role in regulating lysine lactylation and its biological effects. For instance, SIRT2 inhibits lactylation: in gastric cancer, it suppresses the lactylation of METTL16 at K229, and its inhibition enhances METTL16 lactylation to promote cuproptosis, thereby improving the efficacy of copper ionophores (41). SIRT3 acts as a major delactylase, removing lactyl groups from various substrates: in hepatocellular carcinoma (HCC), it reduces the lactylation of CCNE2 at K348 to suppress cell growth and induce apoptosis; in mitochondria, it delactylates PDHA1 and CPT2 to reverse their inactivation and activate oxidative phosphorylation; and in NK cells, it delactylates ROCK1 to restore cytotoxicity by preventing mitochondrial fragmentation (22). SIRT1 mediates the delactylation of PTBP1 in glioma stem cells, attenuating PTBP1’s stability and RNA-binding capacity to inhibit glycolysis and tumorigenesis (21). Additionally, SIRT6 reduces the lactylation of ALKBH5 during viral infections, limiting ALKBH5’s ability to promote IFN-β mRNA biogenesis and antiviral responses (123) (**Figure 5**).

In addition to the well-established roles of p300/CBP and the SIRT family in regulating lactylation, other enzymes such as GCN5 and histone deacetylases (HDACs) also contribute significantly to the lactylation landscape, as discussed in earlier sections (see Sections 2.1 and 2.2). GCN5, a known histone acetyltransferase, has been identified as a lactyltransferase in ovarian cancer, where it catalyzes H3K9la to activate homologous recombination repair genes such as *RAD51* and *BRCA2*, thereby promoting platinum resistance (67). This highlights its dual functionality in both acetylation and lactylation, further complicating the epigenetic regulatory network. HDAC1 and HDAC2, traditionally recognized as deacetylases, also function as delactylases. For instance, in pancreatic ductal adenocarcinoma, HDAC2 downregulation leads to accumulation of H3K18la, activating mitotic checkpoint genes and driving tumor proliferatio (24). This underscores the multifaceted roles of HDACs in erasing lactylation marks, thereby influencing tumorigenesis and therapy response.

Collectively, these findings underscore a broad enzymatic network—including p300/CBP, GCN5, HDACs, and SIRTs—that dynamically regulates lactylation, profoundly impacting tumor progression, metabolic reprogramming, immune modulation, and therapeutic outcomes.

## 8 Clinical application and interventions

While it is true that a significant portion of the findings on lactylation and lactate metabolism in cancer are derived from *in vitro* and animal models, there is growing evidence supporting its clinical relevance. For instance, elevated expression of lactate dehydrogenase A (LDHA) and monocarboxylate transporters (MCT1/4) has been consistently associated with poor prognosis in multiple human cancers, including melanoma (124), glioblastoma (125), and non-small cell lung cancer (NSCLC) (126). Moreover, lactylation modifications—such as H3K18la—have been specifically correlated with aggressive tumor phenotypes and worse patient outcomes in clinical samples, such as in ocular melanoma and clear cell renal cell carcinoma (15). These observations strongly suggest that lactylation-related markers hold promise as potential prognostic biomarkers in human malignancies.

Moving beyond biomarkers, several lactate-targeting therapeutic strategies have already advanced into clinical evaluation. A prime example is the MCT1 inhibitor AZD3965, which has completed a Phase I/II clinical trial (NCT01791595) in patients with advanced solid tumors and lymphomas, demonstrating tolerable pharmacokinetics and on-target effects (127, 128).

Furthermore, advancements in metabolic imaging are facilitating the translation of lactylation research into clinical practice. Techniques like FDG-PET, along with the development of novel lactate-specific probes (e.g., Fila), aim to enable non-invasive monitoring of lactate metabolism dynamics within patients (129, 130). This capability is crucial for both clinical diagnostics and the assessment of therapeutic response, ultimately bridging the gap between laboratory discoveries and patient care.

## 9 Challenges and prospects

Despite significant progress in lactylation research, its clinical translation still faces multiple challenges. At the basic research level, lactylation detection technologies need to be optimized, as the sensitivity of existing mass spectrometry methods is insufficient to capture low-abundance modifications; the identification of specific “readers” is still inadequate, limiting the understanding of downstream effector pathways (131). In addition, the cross-regulatory network between lactylation and other post-translational modifications (such as acetylation, methylation) has not been fully elucidated, and the mechanisms of their synergistic or antagonistic effects need in-depth study (78, 132).

In clinical research, there is a lack of tissue-specific lactylation markers; current studies mostly focus on pan-lactylation or single modification sites, which are difficult to reflect the overall lactylation status of tumors. The off-target effects of existing inhibitors may lead to toxic side effects; for example, non-selective inhibition of the HDAC family may affect the epigenetic regulation of normal cells (106). In addition, lactylation is highly heterogeneous among different tumor types, requiring the development of personalized intervention strategies (133).

Furthermore, the variability among different cancer types indeed poses a significant challenge to the generalizability of lactylation-mediated mechanisms and therapeutic strategies. This heterogeneity stems from differences in metabolic reprogramming patterns, tumor microenvironment composition, oncogenic drivers, and tissue-specific contexts. For instance, while lactylation drives bevacizumab resistance in colorectal cancer through H3K18la-mediated activation of the autophagy gene *RUBCNL*—a process linked to CRC’s hypoxic glycolytic phenotype and crosstalk with homeostatic genes-regulated macrophage polarization. Such cancer-type specificity extends to the expression of lactylation regulators and metabolic dependencies: MCT1 inhibition reduces lactate uptake and HIF1α lactylation to suppress angiogenesis in prostate cancer but shows limited efficacy in pancreatic adenocarcinoma where tumor cells preferentially use MCT4 for lactate export (134). Similarly, SIRT2 inhibition enhances METTL16 lactylation to promote cuproptosis in gastric cancer yet exacerbates ALDOA lactylation to reinforce stemness in liver cancer (41). Therefore, the translational potential of lactylation-targeted therapies must be carefully evaluated within the context of cancer-specific metabolic and epigenetic landscapes, necessitating the development of personalized strategies that account for tumor-type variations.

Future research should focus on the following directions: overcoming the current challenges will pave the way for several exciting clinical applications. First, the development of high-sensitivity, site-specific lactylation detection kits for clinical specimens (e.g., immunohistochemistry assays for H3K18la) could enable routine pathological assessment, guiding prognosis and patient selection for targeted therapies. Second, the tumor-type specificity of lactylation mechanisms necessitates the development of personalized intervention strategies. For instance, MCT1 inhibition might be more effective in prostate cancer, while targeting SIRT2 may be a strategy for gastric cancer but could be contraindicated in liver cancer. Finally, the most promising near-term application lies in combination therapies. Given lactylation’s role in driving immunosuppression and therapy resistance, combining lactylation inhibitors (e.g., LDHA inhibitors, delactylase agonists) with immune checkpoint blockade, chemotherapy, or targeted agents represents a rational strategy to overcome resistance and improve outcomes, particularly in ‘immune-cold’ and treatment-refractory tumors.

## 10 Conclusion

As a crucial nexus between metabolism and epigenetics, lactylation offers a novel perspective for cancer therapy. With the deepening of mechanistic insights and ongoing technological innovations, therapeutic strategies targeting lactylation hold significant promise as a breakthrough in overcoming current therapeutic limitations, offering new avenues for biomarker development and combination therapies and new hope for improving cancer patient prognosis.
